# Prevalence of Methicillin-Resistant *S. aureus*, Extended-Spectrum β-Lactamase-Producing *E. coli*, and Vancomycin-Resistant *E. faecium* in the Production Environment and Among Workers in Low-Capacity Slaughterhouses in Poland

**DOI:** 10.3390/antibiotics14121200

**Published:** 2025-11-28

**Authors:** Anna Ławniczek-Wałczyk, Marcin Cyprowski, Małgorzata Gołofit-Szymczak, Rafał L. Górny

**Affiliations:** Laboratory of Biohazards, Department of Chemical, Aerosol and Biological Hazards, Central Institute for Labour Protection—National Research Institute (CIOP-PIB), 16 Czerniakowska Street, 00-701 Warsaw, Poland

**Keywords:** MRSA, ESBL, VRE, slaughterhouses, antimicrobial resistance, biofilm

## Abstract

**Background:** Small-scale food animal production is common worldwide but often underestimated as a source of antimicrobial resistance. This study aimed to determine the prevalence of MRSA and VRE-*E. faecium*, and ESBL-*E. coli* bacteria among workers and within the production environment of low-capacity slaughterhouses, as well as to analyze the antimicrobial resistance patterns of these bacteria and their ability to form biofilms. **Methods:** The measurements were carried out in three low-capacity slaughterhouses in Poland. Bioaerosol samples, swabs from the production environment fomite and carcasses, meat samples, and swabs from workers’ hands and nostrils were taken. The strains’ susceptibility to antibiotics was assessed using the disk diffusion method, and their biofilm-forming potential was assessed using the microplate method. Isolates were also tested for the presence of genes related to biofilm formation and resistance to antiseptics. **Results:** In this study, 13.8%, 20.5%, and 14.9% of the samples (*n* = 268) were positive for MRSA, ESBL-*E. coli*, and VRE-*E. faecium*, respectively, with the highest detection rates on pork carcasses and surfaces. MRSA and ESBL-*E. coli* bacteria were also detected in swabs from workers’ hands and nasal swabs, and in bioaerosol samples. Most isolates revealed multidrug resistance, including 89% of MRSA, 76% of ESBL-*E. coli*, and 83% of VRE-*E. faecium*. The majority of them were also capable of biofilm formation—81%, 65%, and 75%, respectively—emphasizing their survival capabilities in slaughterhouse environments. **Conclusions:** The slaughterhouse workers are regularly exposed to antibiotic-resistant bacteria such as MRSA, ESBL-*E. coli*, and VRE-*E. faecium*. To reduce these risks, it is essential for small slaughterhouses to strictly follow hygiene protocols, enhance the separation between clean and contaminated areas, improve ventilation, and ensure the use of protective measures.

## 1. Introduction

The growing resistance of bacteria to antimicrobial agents poses a serious global threat, already causing over a million deaths annually and affecting public health, food safety, as well as the health of workers [1,2]. The meat industry remains the largest consumer of antibiotics, where they are widely used in intensive livestock farming—not only for therapeutic and prophylactic purposes, but also as feed additives to promote growth [3,4,5]. For the first time, all 27 Member States of the European Union, together with Iceland and Norway, have provided integrated data on both the sale and use of antimicrobials in animals. The data show that as much as 98.4% of the products sold are intended for farm animals, and only 1.6% for companion animals [6]. In the context of the EU’s Farm to Fork Strategy, which aims to reduce antibiotic consumption by 50% by 2030, the current level (88.5 mg/PCU in 2023) means that approximately half of the target has been achieved. However, there are still significant differences between Member States, which highlights the need for further coordinated action at the European level [5,6]. From a food safety perspective, the problem of antibiotic resistance mainly concerns three groups of microorganisms isolated from food products: *Staphylococcus* spp. (including MRSA strains), ESBL-producing *Enterobacteriaceae*, and vancomycin-resistant *Enterococcus* spp. (VRE) [5]. These antimicrobial-resistant strains, as well as other bacterial pathogens present in both farming environments and meat production facilities, may persist on carcasses, processing surfaces, and in bioaerosols, and can also colonize workers, creating a transmission interface between animals, food products, and humans [7,8,9,10,11,12,13,14]. The duration and intensity of direct contact with livestock [15,16], as well as the prevalence of MRSA, ESBL, and VRE positive animals on farms, have been strongly linked to colonization and infection in humans [17,18,19,20]. An increased risk of infection with drug-resistant microorganisms is confirmed among workers who have daily contact with live animals. It has been confirmed that livestock animals such as pigs, chickens, and cattle can frequently harbor *Enterobacteriaceae*-producing extended-spectrum β-lactamases (ESBLs) [21,22,23,24,25], methicillin-resistant *S. aureus* (MRSA) [26,27,28,29], and vancomycin-resistant enterococci (VRE) [20,30,31,32]. An additional challenge lies in their ability to form biofilms on a wide variety of production-related surfaces [20,31]. Biofilm-associated cells are significantly more tolerant to antimicrobial agents than their planktonic counterparts, and mature biofilm structures are extremely difficult to eradicate [33,34]. This property that enables long-term persistence in slaughterhouse and processing environments increases the risk of both cross-contamination and occupational exposure [19,31,35,36,37,38,39]. Importantly, multidrug resistance (MDR), frequently observed among MRSA, ESBL-producing *E. coli*, and VRE, exacerbates these risks and has been globally identified as one of the greatest threats to human and public health. The report Antibiotic Resistance: One Health One World Outlook emphasizes that antimicrobial resistance is a cross-sectoral challenge with direct links between agricultural practices, environmental dissemination, and human health [40]. Resistant bacteria in the food chain not only undermine the effectiveness of antimicrobial therapy but also threaten the sustainability of safe food production [41]. While industrial farms and slaughterhouses are well-studied for worker exposure to harmful biological agents [8,9,18,20,26,28,42,43,44], we still know very little about pathogen transmission pathways in low-capacity slaughterhouses producing traditional meat products. Small-scale food animal production is common worldwide but often underestimated as a source of antimicrobial resistance (AMR). The frequent use of antimicrobials in such systems promotes the emergence of resistant bacteria, which can spread to humans during slaughter and processing, especially in facilities with limited hygiene controls [45]. These pathogens pose risks to workers and consumers alike, and their circulation beyond local settings contributes to the wider dissemination of AMR. Effective monitoring and targeted interventions in smallholder farms and abattoirs are therefore essential elements of the One Health approach [40].

The present study was undertaken to investigate the occurrence of MRSA, vancomycin-resistant *Enterococcus faecium* (VRE-*E. faecium*), and ESBL-producing *Escherichia coli* (ESBL-*E. coli*) in low-capacity slaughterhouses in Poland. The research assessed antimicrobial susceptibility profiles, biofilm-forming capacity, and the presence of virulence genes in these isolates. In addition, the study aimed to characterize hand and nasal carriage of MRSA, VRE-*E. faecium*, and ESBL-*E. coli* among slaughterhouse workers, providing insights into both environmental contamination and occupational exposure risks.

## 2. Results

### 2.1. General Characteristics of the Studied Population of Workers

A total of 37 participants from three low-capacity slaughterhouses were enrolled in this study. Workers were divided into two groups; the first included butchers who slaughtered animals, eviscerated carcasses, cut and processed fresh carcasses, produced sausages, and hams. The second group consisted of non-production workers who were not directly involved in meat production; among them were supervisory workers and salespeople (Table 1). Men predominated among the participants in the study, accounting for 78% of the respondents. The average age of workers was 41 years (20–63 years).

### 2.2. Occurrence of ESBL-E. coli, MRSA, and VRE-E. faecium

The distribution of ESBL-*E. coli*, MRSA, VRE-*E. faecium* and antibiotic-sensitive isolates—non-extended-spectrum beta-lactamase-producing *E. coli* (non-ESBL-*E. coli*), methicillin-sensitive *S. aureus* (MSSA), and vancomycin-susceptible *E. faecium* (VSE-*E. faecium*)—collected in the studied low-capacity slaughterhouses are presented in Table 2 and Table 3.

Out of 268 tested samples, 180 (67%) were *E. coli* positive. Based on the disk diffusion method for the determination of ESBL-producing phenotypes and the PCR test, 55 of the *E. coli* isolates were ESBL producers, and 125 were non-ESBL-*E. coli*. The highest isolation rate of ESBL-*E. coli* was observed in pork carcasses (67%), bioaerosols (43%), and in surface samples (32%). Considering the location of the sampling, the highest percentage of ESBL-positive samples was noted in slaughter and in meat cutting sections, where ESBL-*E. coli* was particularly prevalent in bioaerosols samples (75% and 50%, respectively), swabs from hooks and trays (67%), floors (38 and 13%), and meat containers (38%). The analysis showed that 30% of slaughterhouse workers (9 out of 30) carried ESBL-*E. coli*, compared with 14% (1 out of 7) among non-production staff. ESBL-*E. coli* was recovered from butchers’ hands (six isolates) and nostrils (three isolates), and from one nasal sample obtained from a non-production worker. However, the difference in carriage rates between the two groups was not statistically significant. In this study, 57% (154/268) of the samples were positive for *S. aureus*, and 37 isolates were phenotypically and genetically identified as MRSA, and 117 as MSSA. The highest MRSA isolation rate was noted in pork carcasses (70%) and in swabs from surfaces (26%). Taking into account the location of sampling, the highest percentage of MRSA-positive samples was noted in slaughter and in meat processing sections, where MRSA were often recovered from swabs from stuffer machines (37.5%), hooks and trays (33%), as well as conveyors (25%). MRSA bacteria were also detected in swabs taken from butchers (one from hands and one from nasal swabs) and non-production workers (one isolate from hands). A total of 40 out of 141 detected *E. faecium* isolates were identified as VRE-*E. faecium*, while 81 were identified as VSE-*E. faecium*. The VRE-*E. faecium* isolates were most often isolated from pork carcasses (42%) and surface samples (39%). The analysis showed that 17% of slaughterhouse workers (five out of 30) carried VRE-*E. faecium*, whereas the carriage rate among non-production workers was 14% (one out of seven). No VRE-*E. faecium* isolates were detected in nasal swabs. Samples from ready-to-eat products did not contain any ESBL-*E. coli*, MRSA, and VRE-*E. faecium* isolates.

### 2.3. Characteristics of ESBL-E. coli Isolates

#### 2.3.1. Antibiotic Resistance Profiles of ESBL-*E. coli* Isolates

**Figure 1:**
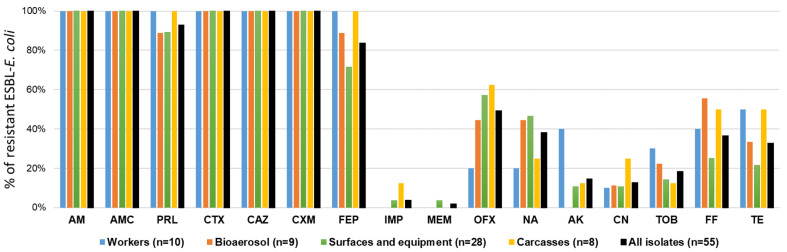
Antibiotic resistance of ESBL-*E. coli* collected from tested low-capacity slaughterhouses. AM (ampicillin), AMC (amoxicillin–clavulanic acid), PRL (piperacillin), CTX (cefotaxime), CAZ (ceftazidime), CXM (cefuroxime), FEP (cefepime), IMP (imipenem), MEM (meropenem), OFX (ofloxacin), NA (nalidixic acid), AK (amikacin), CN (gentamicin), TOB (tobramycin), FF (nitrofurantoin), and TE (tetracycline).

**Figure 2:**
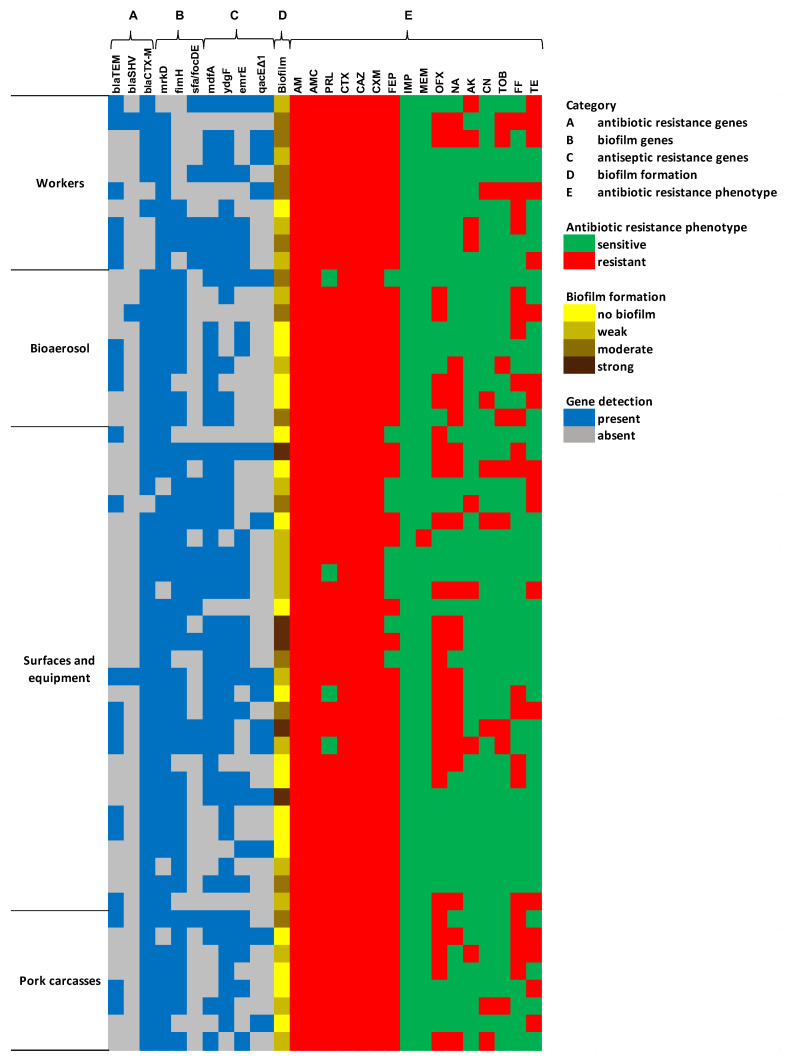
The heat map of the drug resistance profiles, genotypic and phenotypic characterization of ESBL-*E. coli* isolates. AM (ampicillin), AMC (amoxicillin–clavulanic acid), PRL (piperacillin), CTX (cefotaxime), CAZ (ceftazidime), CXM (cefuroxime), FEP (cefepime), IMP (imipenem), MEM (meropenem), OFX (ofloxacin), NA (nalidixic acid), AK (amikacin), CN (gentamicin), TOB (tobramycin), FF (nitrofurantoin), and TE (tetracycline).

Antibiotic resistance phenotypes of ESBL-*E. coli* isolates are shown in Figure 1 and Figure 2, and Table S1. The ESBL-*E. coli* isolates were highly resistant to most of the tested β-lactam antibiotics, including cefotaxime (100%, 55/55), cefuroxime (100%), ceftazidime (100%), cefepime (84%), as well as others antibiotics, such as ampicillin (100%), amoxicillin–clavulanic acid (100%), ofloxacin (49%), nalidixic acid (38%), fosfomycin (36%), and tetracycline (33%). The isolates were susceptible to tested carbapenems, including meropenem (98%) and imipenem (96%), and to aminoglycosides (respectively, 86% and 87% for amikacin and gentamycin). The comparison of resistance profiles between isolates showed no major differences in β-lactam resistance. However, minor variation was observed for fluoroquinolone resistance. Only 20% of worker isolates were resistant to ofloxacin and nalidixic acid, compared with 57% and 62% of isolates from surfaces and carcasses, respectively. Out of 55 ESBL-*E coli* isolates, 42 (76%) isolates were determined as MDR (resistant to ≥3 classes of antimicrobials), and their multiple antibiotic resistance index (MARI) ranged from 0.5 to 0.81. The prevalence of MDR ESBL-*E. coli* was particularly noticeable in carcass samples, where they accounted for 100% of all isolates. A total of 33 resistance profiles of ESBL-*E. coli* isolates were identified (Table S1). The most common spectrum of resistance was AM-AMC-PRL-CTX-CAZ-CXM-FEP (18%, 10/55), AMC-PRL-CTX-CAZ-CXM-FEP-OFX-NA-FF-TE (7%, 4/55), and AM-AMC-PRL-CTX-CAZ-CXM-FEP-OFX-FF (5.5%, 3/55).

#### 2.3.2. Genetic Characterization and Biofilm Formation Potential of ESBL-*E. coli*

**Figure 3:**
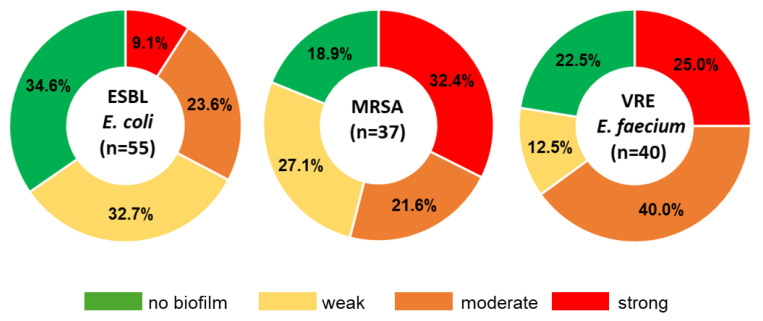
Biofilm-forming ability of ESBL-*E. coli*, MRSA, and VRE-*E. faecium* isolates.

Of the 55 ESBL-*E.coli* isolates, each had at least one of the ESBL genes tested (Figure 2). Among them, 91% (50/55), 38% (21/55), and 5.5% (3/55) carried *blaCTX-M*, *blaTEM*, and *blaSHV* genes, respectively. The distribution of detected ESBL genotypes among isolates was as follows: *blaCTX-M* (60%, 33/55), *blaTEM*-*blaCTX-M* (25%, 14/55), *blaTEM* (9%, 5/55), and *blaTEM*-*blaSHV*-*blaCTX-M* (5%, 3/55). Biofilm analysis showed that 65% of ESBL-*E. coli* isolates were capable of biofilm formation, of which 9%, 24%, and 33% were classified as strong, moderate, and weak biofilm producers (Figure 3).

**Figure 4:**
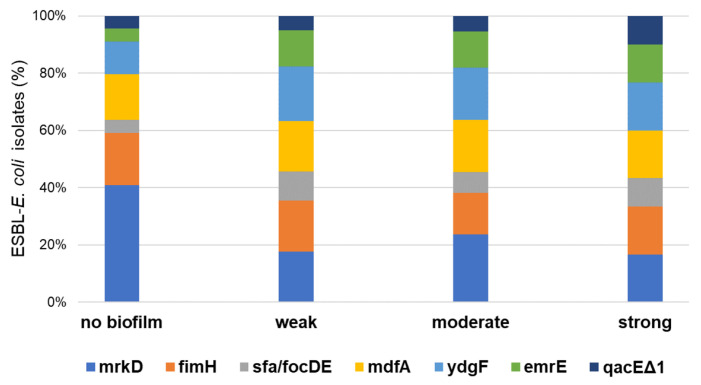
Distribution of biofilm factor-encoding genes and antiseptic-resistance genes among ESBL-*E. coli* isolates with different biofilm-forming abilities.

Figure 4 presents the prevalence of biofilm-related (*fimH*, *sfa/focDE*, *mrkD*) and antiseptic-resistance genes (*mdfA*, *ydgF*, *emrE*, and *qacEΔ1*) detected among ESBL-*E. coli* isolates. The most common biofilm factors encoding genes were *mrkD* (91%, 50/55), *fimH* (76%, 42/55), and *sfa/focDE* (31%, 17/55). In the current study, four antiseptic-resistance genes, including *mdfA*, *ydgF*, *emrE*, and *qacEΔ1* were found in 91% (50/55) of the ESBL-*E. coli* isolates. The *ydgF* (76%, 39/55) and *mdfA* (71%, 39/55) genes were the most common in the tested strains, followed by the *emrE* (49%, 27/55) and *qacEΔ1* (27%, 15/55) genes. A significant association was observed between the occurrence of MDR and the biofilm-forming abilities of *E. coli* (*p* < 0.00001). Moreover, the presence of *fimH*, *sfa/focDE*, *mdfA*, *ydgF*, and *emrE* genes was higher in the group of biofilm producers than in the non-biofilm producers (*p* < 0.05).

### 2.4. Characteristics of MRSA Isolates

#### 2.4.1. Antibiotic Resistance Patterns of MRSA Isolates

**Figure 5:**
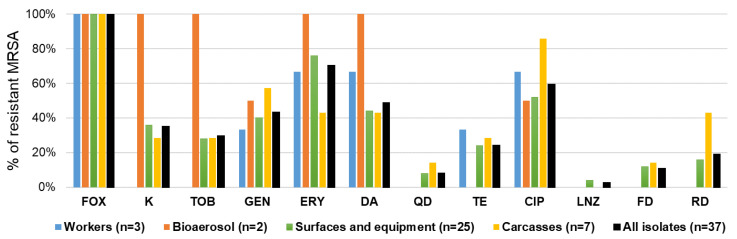
Antibiotic resistance of MRSA strains collected from tested low-capacity slaughterhouses. FOX (cefoxitin), K (kanamycin), TOB (tobramycin), GEN (gentamicin), ERY (erythromycin), DA (clindamycin), QD (quinupristin–dalfopristin), TE (tetracycline), CIP (ciprofloxacin), LNZ (linezolid), FD (fosfomycin), and RD (rifampicin).

**Figure 6:**
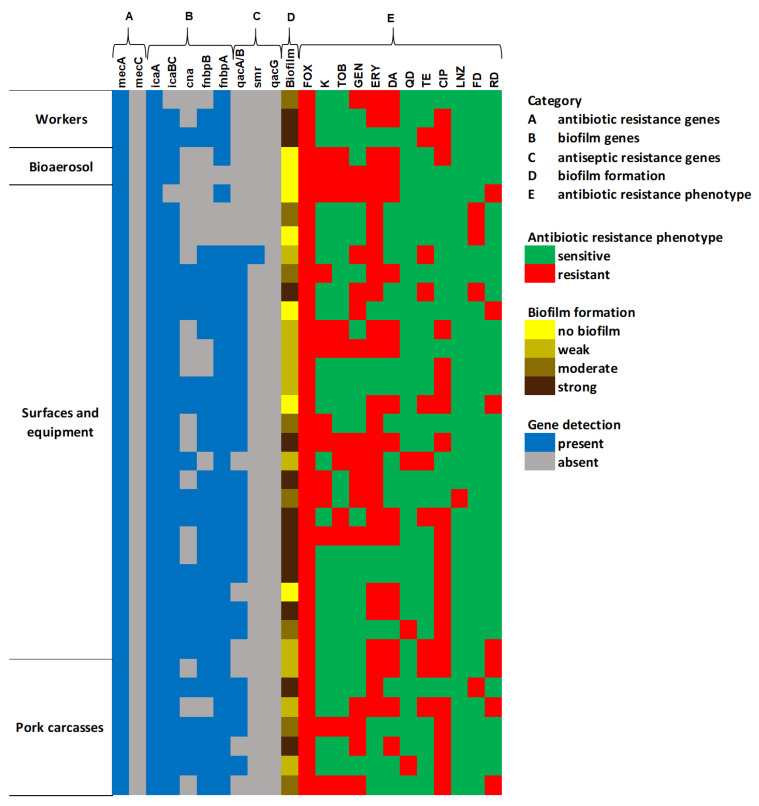
The heat map of the drug resistance profiles, genotypic and phenotypic characterization of MRSA isolates. FOX (cefoxitin), K (kanamycin), TOB (tobramycin), GEN (gentamicin), ERY (erythromycin), DA (clindamycin), QD (quinupristin–dalfopristin), TE (tetracycline), CIP (ciprofloxacin), LNZ (linezolid), FD (fosfomycin), and RD (rifampicin).

Antibiotic resistance phenotypes of MRSA isolates were illustrated in Figure 5 and Figure 6, and in Table S2. The majority of MRSA isolates showed high resistance rates to erythromycin (70.3%), ciprofloxacin (59.5%), clindamycin (48.6%), gentamicin (43.2%), and kanamycin (35.1%). The isolated strains were particularly susceptible to linezolid (97%). MRSA isolates collected from bioaerosols demonstrated the widest range of resistance among all tested groups. All (100%) of the isolates were resistant to kanamycin, tobramycin, erythromycin, and clindamycin. Additionally, 50% of these isolates were resistant to gentamicin and ciprofloxacin. However, there was no observed resistance to linezolid or rifampicin. In contrast, MRSA isolates from surfaces exhibited significant resistance to erythromycin (76%), ciprofloxacin (52%), gentamicin (40%), and clindamycin (44%). Isolates obtained from carcasses displayed particularly high resistance to ciprofloxacin (86%), along with gentamicin (57%), and a modest resistance to erythromycin (43%), clindamycin (43%), tetracycline (29%), and kanamycin (29%). All strains were sensitive to linezolid. MRSA isolates taken from workers also showed considerable resistance, with 67% resistant to erythromycin, clindamycin, and ciprofloxacin.

Among 37 MRSA isolates, 89% were classified as MDR (resistant to ≥3 classes of antimicrobials), and their multiple antibiotic resistance index (MARI) ranged from 0.25 to 0.58. The prevalence of MDR MRSA was particularly noticeable in the carcasses and meat samples, where they accounted for 100% of all isolates and presented high resistance to CIP (86%) and Gen (57%). A total of 24 resistance profiles were identified. The most common spectrum of resistance was FOX-CIP (11%), FOX-ERY-FD (8%), FOX-ERY-DA-TE-CIP-RD (8%), and FOX-ERY-DA-CIP (8%) (Table S2).

#### 2.4.2. Genetic Characterization and Biofilm Formation Potential of MRSA

**Figure 7:**
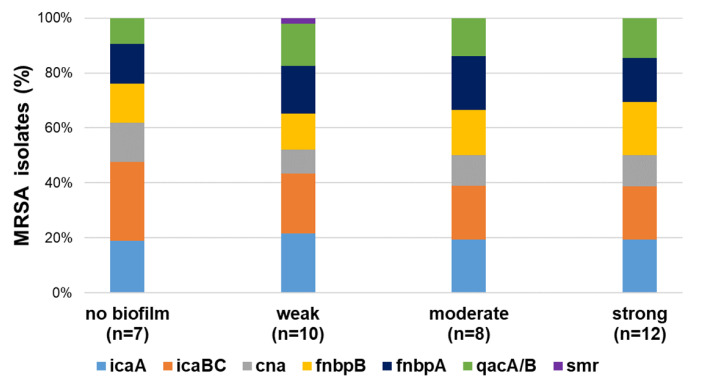
Distribution of biofilm factor-encoding genes and antiseptic-resistance genes among MRSA isolates with different biofilm-forming abilities.

Genetic characterization and biofilm formation potential of MRSA isolates are presented on a heatmap in Figure 6. A representative *mecA* gene was confirmed in 37 MRSA isolates. Among them, 89% (33/37), 94% (35/37), 48% (18/55), 73% (27/37), and 76% (28/37) carried *icaA*, *icaBC*, *cna*, *fnbpB*, and *fnbpA* genes, respectively. The most common antiseptic-resistance gene detected in MRSA was *qacA/B* (62%, 23/37). The *smr* gene was present only in one isolate, and no MRSA isolates harbor the *qacG* gene. Biofilm formation assay showed that 81% of MRSA isolates were capable of biofilm formation, of which 32.4%, 21.6%, and 27% were classified as strong, moderate, and weak biofilm producers (Figure 3). Figure 7 presents the prevalence of biofilm-related (*icaA*, *icaBC*, *cna*, *fnbpB*, and *fnbpA*) and antiseptic-resistance genes (*qacA/B*, *smr*, and *qacG*) detected among MRSA isolates. The presence of *icaA*, *icaBC*, *cna*, *fnbA*, *fnbB*, and *qacA/B* genes was higher in the group of biofilm producers than in the non-biofilm producers, but this association was statistically significant only for *fnbA*, *fnbB*, and *qacA/B* genes (*p* < 0.05). There was no significant association between the presence of MDR and the biofilm formation ability in MRSA isolates.

### 2.5. Characteristics of VRE-E. faecium Isolates

#### 2.5.1. Antibiotic Resistance Profiles of VRE-*E. faecium* Isolates

**Figure 8:**
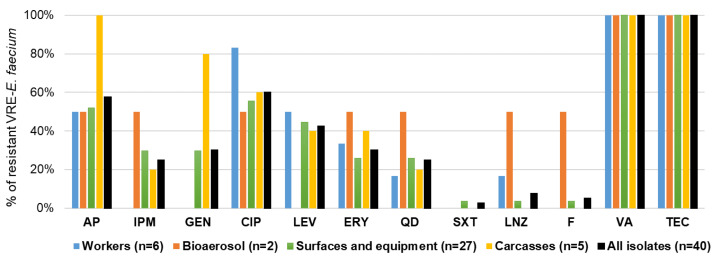
Antibiotic resistance of VRE-*E. faecium* collected from tested low-capacity slaughterhouses. AP (ampicillin), IPM (imipenem), GEN (gentamicin), CIP (ciprofloxacin), LEV (levofloxacin), ERY (erythromycin), QD (quinupristin–dalfopristin), SXT (trimethoprim–sulfamethoxazole), LNZ (linezolid), F (nitrofurantoin), VA (vancomycin), and TEC (teicoplanin).

**Figure 9:**
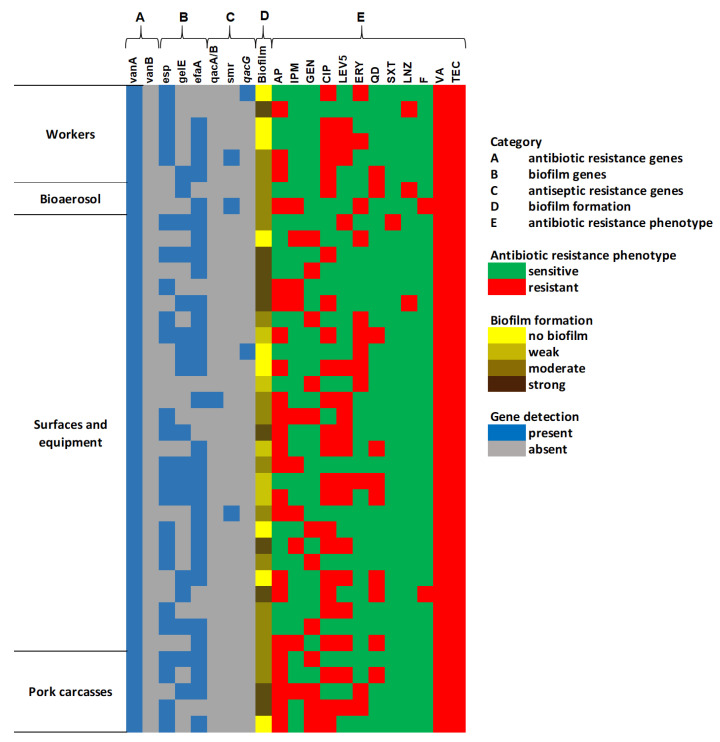
The heat map of the drug resistance profiles, genotypic and phenotypic characterization of VRE-*E. faecium* isolates. AP (ampicillin), IPM (imipenem), GEN (gentamicin), CIP (ciprofloxacin), LEV (levofloxacin), ERY (erythromycin), QD (quinupristin–dalfopristin), SXT (trimethoprim–sulfamethoxazole), LNZ (linezolid), F (nitrofurantoin), VA (vancomycin), and TEC (teicoplanin).

Antibiotic resistance phenotypes of VRE-*E. faecium* isolates were illustrated in Figure 8 and Figure 9, and Table S3. The majority of VRE isolates showed high resistance rates to vancomycin (100%), teicoplanin (100%), ciprofloxacin (60%), ampicillin (58%), and levofloxacin (43%). The isolated strains were particularly susceptible to trimethoprim–sulfamethoxazole (98%), nitrofurantoin (95%), and linezolid (93%). Isolates obtained from workers exhibited predominant resistance to ciprofloxacin (83%), ampicillin (50%), and levofloxacin (50%). VRE isolates recovered from bioaerosol samples displayed a broad resistance pattern, with comparable frequencies (50%) of resistance to ampicillin, imipenem, erythromycin, quinupristin–dalfopristin, linezolid, and nitrofurantoin. Surface isolates frequently showed resistance to ciprofloxacin (56%), ampicillin (52%), and levofloxacin (44%). In contrast, isolates from carcasses exhibited the broadest resistance spectrum, characterized by high resistance to ampicillin (100%), gentamicin (80%), and ciprofloxacin (60%), and lower resistance to levofloxacin and erythromycin (40% each). Among 40 VRE isolates, 83% were classified as MDR, and their MARI ranged from 0.33 to 0.58. The prevalence of multidrug-resistant VRE-*E. faecium* was particularly high among isolates collected from bioaerosol samples, carcasses, and workers, accounting for 100%, 83%, and 83% of isolates, respectively. A total of 29 resistance profiles were identified (Table S3). The most common spectrum of resistance was AP-CIP-LEV-QD-VA-TEC-(3/40, 7.5%), AP-CIP-LEV-VA-TEC-(3/40, 7.5%), AP-IPM-VA-TEC-(3/40, 7.5%), and GEN-VA-TEC-(3/40, 7.5%).

#### 2.5.2. Genetic Characterization and Biofilm Formation Potential of VRE Isolates

**Figure 10:**
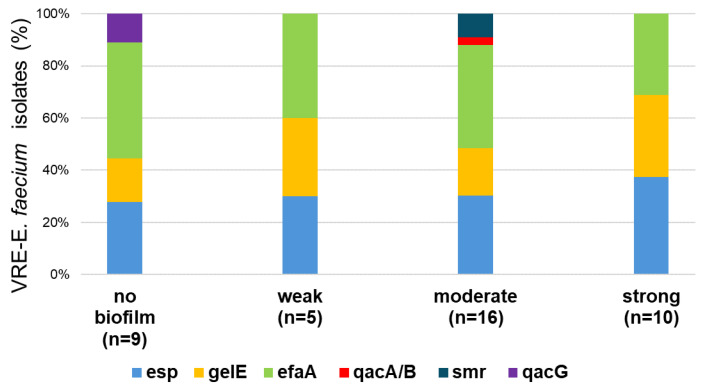
Distribution of biofilm factor-encoding genes and antiseptic-resistance genes among VRE-*E. faecium* isolates with different biofilm-forming abilities.

Genetic characterization and biofilm formation potential of VRE-*E. faecium* isolates are presented on a heatmap in Figure 9 and Figure 10. All 40 isolates of VRE-*E. faecium* were found to carry the *vanA* gene, while none of the isolates carried the *vanB* gene (Figure 9). Among these, the *efaA* gene was the most commonly detected, with a prevalence rate of 75% (30 out of 40 isolates), followed by *esp* at 60% (24 out of 40), and *gelE* at 42.5% (17 out of 40). A small percentage of the studied VRE isolates carried genes associated with antiseptic resistance. Specifically, the *smr* gene was present in three isolates only, the *qacG* gene was found in two isolates, and the *qacA/B* gene was detected in one isolate (Figure 10). Biofilm analysis revealed that 77.5% of the VRE-*E. faecium* isolates were capable of biofilm formation (Figure 3). Among these, 25% were classified as strong producers, 40% as moderate producers, and 13% as weak producers. Furthermore, no significant association was found between their biofilm-forming abilities, the occurrence of multidrug resistance, and the presence of virulence or antiseptic resistance genes.

## 3. Discussion

Maintaining the high quality of meat is a very difficult task. At almost every stage of its production (from the moment of animal breeding to slaughtering to processing and distribution), there are many environmental factors that may negatively affect its microbiological purity and health safety [41,42,46]. However, little information is available concerning the prevalence of antibiotic-resistant bacteria in low-capacity slaughterhouses. Knowledge about the main pathways of microbiological contamination in meat production is essential to update the hygiene plan of a given plant and to guide the cleaning and disinfection processes.

Antimicrobial-resistant bacteria detected in slaughterhouses, particularly MRSA, ESBL-*E. coli*, and VRE-*E. faecium*, represent overlapping risks for food contamination, environmental persistence, and occupational exposure. Abattoirs can function as transmission nodes where resistant organisms circulate between animals, carcasses, equipment, and workers, underscoring the importance of control measures specifically tailored to small-capacity facilities [47,48]. The present study demonstrated that more than half of the *S. aureus* isolates recovered from low-capacity slaughterhouses were methicillin-resistant (MRSA), with the highest prevalence observed on pork carcasses (70%) and processing surfaces (19%). The detection of MRSA on workers’ hands (10%) and in their nostrils (3%) suggests occupational exposure. However, the prevalence observed in our study was lower than that reported in several previously studied high-exposure groups. For instance, Cuny et al. (2009) documented nasal colonization in 45% of veterinarians working with pig farms and in 9% of their non-exposed household members [49]. The lower rates observed in our study likely reflect both the smaller sample size and the specific characteristics of low-capacity slaughterhouses. De Jonge et al. (2010) [50] and Cuny et al. (2019) [51] noted that the risk of LA-MRSA colonization through handling raw meat appears to be very low among professionals working in institutional kitchens and butcheries. This is attributed to lower levels of bacterial contamination in such environments and the application of proper hygiene practices, which are unlikely to result in nasal colonization solely from product handling. On the other hand, other studies have shown that the consumption of poultry meat contaminated with MRSA may increase the risk of acquiring nasal colonization [52]. This observation is in good agreement with reports from European and Asian abattoirs, where MRSA—often livestock-associated lineages such as CC398—has been identified in pigs, carcasses, environments, and workers. Occupational studies have further shown that personnel working in direct exposure in the so-called dirty part of the slaughterhouse/abattoir (lairage, scalding/dehairing) are at increased risk of being a carrier [27,47,53,54]. Since MRSA carriage may elevate the risk of complications during hospitalization [55], our findings emphasize the need to develop preventive measures for professionals who are in contact with livestock and raw meat. In this context, further public health efforts are of major importance, including the establishment of monitoring systems to enable trend analysis of the risk of MRSA transmission from animals to humans. Resistance profiles observed here (frequent macrolide, fluoroquinolone, and aminoglycoside resistance with preserved linezolid susceptibility) reflect the multidrug-resistant phenotype typical for LA-MRSA reservoirs and are consistent with prior abattoir-linked studies [56]. Regarding the presence of genes encoding *S. aureus* biofilm-associated adhesins (*icaA/icaBC*, *fnbA*, *fnbB*, *cna*), which were frequently detected among the analyzed MRSA isolates, the results of this study are consistent with findings reported in recent research. These factors are necessary for intercellular adhesion and are associated with both slime and biofilm multilayer formation in *S. aureus* [57,58,59]. The high prevalence of biofilm production (81%), including 27% strong biofilm formers, is consistent with observations by Silva et al. (2021) [58], Vergara et al. (2017) [60], who found similar biofilm capabilities among food-derived MRSA. Biofilm-related persistence has also been described by Pereyra et al. (2016) [61], demonstrating that *S. aureus* with fnb and ica genes exhibits enhanced adhesion. However, they emphasized that adhesion and internalization depend primarily on the overexpression of these genes during host-cell contact rather than on their mere presence. The results of this study are also consistent with findings reported in recent observations of food chain studies demonstrating strong biofilm capacity of *S. aureus*/MRSA on steel and plastic surfaces, which hampers removal by routine sanitation [57,58,59]. Of particular note is the frequent detection of *qacA/B* among MRSA isolates (62%). Although these antiseptic efflux genes are primarily described in healthcare-associated MRSA [62,63], they have been associated with reduced susceptibility to quaternary ammonium compounds and chlorhexidine, suggesting that suboptimal use of disinfectants may inadvertently select for persistence in biofilm-prone environments [64,65]. Moreover, Sultan and Ahmed (2022) [63] reported that the frequencies of *qacA/B* and *smr* genes were significantly higher among MRSA than MSSA isolates and in the group of MDR than in non-MDR isolates. However, the presence of antiseptic resistance genes does not necessarily induce tolerance to disinfectants, as the gene could be silent and not expressed by the bacteria [66]. In summary, these results highlight the need for biofilm-targeted interventions and careful use of biocides (cationic, e.g., quaternary ammonium compounds) in small slaughterhouses, particularly at sites such as stuffer machines, hooks, trays, and conveyors, where MRSA was most frequently detected.

In this study, ESBL-producing *E. coli* was recovered from 31% of all *E. coli*-positive samples, with the highest prevalence found on carcasses (67%), in bioaerosols (43%), and on high-contact surfaces (32%). This is consistent with earlier investigations showing that slaughterhouse air and equipment represent key drivers for the dissemination of ESBL-producing *Enterobacteriaceae*. Pigs may acquire ESBL-producing *E. coli* at different stages, including farming conditions, trading points, transport vehicles, or through contact with infected animals [67,68]. In slaughterhouses, pig carcasses can become contaminated with ESBL and other AMR bacteria during evisceration and subsequent processing [69]. Furthermore, food processing environments and food handlers act as additional reservoirs and vectors for the spread of ESBL-producing bacteria [10,22,70,71,72,73]. Observed resistance profiles of isolates showed complete resistance to third- and fourth-generation cephalosporins and aminopenicillins, but susceptibility to carbapenems and aminoglycosides. This mirrors global surveillance data, where ESBL-*E. coli* remains sensitive to carbapenems but resistant to β-lactams [74]. Extended-spectrum β-lactamases represent a clinically significant group of enzymes encoded by genes located on mobile genetic elements, which facilitate their transfer across bacterial species. Alongside AmpC cephalosporinases and carbapenemases, ESBLs are among the most critical β-lactamase families contributing to the global antimicrobial resistance crisis [75,76]. The most extensively described ESBL families include TEM-, SHV-, and CTX-M-type enzymes. Historically, TEM and SHV variants predominated in clinical isolates, particularly within *Klebsiella pneumoniae*. However, the widespread use of third-generation cephalosporins has driven a rapid expansion of CTX-M-type ESBLs, which have largely replaced TEM and SHV enzymes as the dominant variants [75,77,78]. Observed in this study, the predominance of *blaCTX-M* (91%), followed by *blaTEM* and *blaSHV*, reflects the global dominance of CTX-M-type ESBLs in animal and human reservoirs [48,74,77,78]. In a study conducted by Dahms et al. (2015), CTX-M was also the most prevalent β-lactamase in tested cattle, pig, and farm workers’ fecal samples, while SHV predominated only in samples from poultry [79]. A high proportion of detected ESBL-*E.coli* isolates (76%) were MDR, comparable to reports from pig production chains in Germany [80], Brazil [81], India [82], and occupational studies in workers, where slaughterhouse employees dehairing or eviscerating the animals showed higher carriage rates of MDR ESBL-*E. coli* than non-exposed individuals [17,80]. Occupational studies further indicate that workers involved in evisceration are at particularly high risk of ESBL carriage compared to those working in cold-room or packaging areas, supporting the role of task-specific exposure. Martínez-Álvarez et al. (2024) [83] demonstrated that the pig nasal cavity may serve as a reservoir facilitating the dissemination of ESBL-producing *E. coli* and mcr-mediated colistin resistance. Other whole genome studies confirm that pigs and workers can share identical ESBL genotypes, underlining the bidirectional transmission potential at the human–animal interface [84]. At the farm level, transmission may also occur via inhalation of stable air or dust particles, which can serve as carriers of ESBL-producing bacteria [23]. This highlights the need for intensified monitoring, particularly in intensive farming systems where close animal contact increases the likelihood of ESBL resistance spread. A significant association was observed between the occurrence of MDR as well as tested genes (*fimH*, *sfa/focDE*, *mdfA*, *ydgF*, and *emrE*) and the biofilm-forming abilities. Frequent detection of biofilm-associated genes (*fimH*, *sfa/focDE*, *mrkD*) and disinfectant tolerance determinants (*mdfA*, *ydgF*, *emrE*, and *qacEΔ1*) echoes reports about slaughterhouse wastewater and surrounding environments. Such genetic co-occurrence suggests that floor drains and moist equipment surfaces act as reservoirs where biofilm facilitates horizontal gene transfer and promotes persistence under cleaning stress [80,85,86]. Overall, these results reinforce the importance of engineering and operational measures to reduce aerosolization during evisceration and cutting, combined with biofilm-active sanitation of drains and conveyors, and supplemented by worker-level protective measures such as PPE and hygiene protocols.

In this study, VRE bacteria were detected on carcasses, food-contact surfaces, and workers’ hands, but not in nasal swabs, indicating that contamination is more likely contact-mediated rather than airborne. This finding is consistent with reports describing the persistence of *Enterococcus* spp. along pork chains and in retail meats, with *vanA* as the predominant resistance determinant [43,87,88]. The prevalence of VRE in livestock has historically been linked to the use of avoparcin, a glycopeptide antibiotic formerly employed as a growth promoter, with farm workers exposed to this compound showing higher colonization rates [89,90]. Even after the ban on avoparcin use across Europe in the late 1990s, VRE continues to be isolated from a variety of food and animal sources, highlighting a persistent public health concern. Since VRE can colonize the entire food chain, it is essential to monitor its prevalence and investigate potential transmission pathways between animal reservoirs and humans in order to develop effective mitigation strategies [91,92,93]. European studies have long emphasized food chains as reservoirs for VRE and have highlighted the multidrug resistance and strong biofilm-forming capacity [20,31,94]. In this study, almost 85% of all VRE-*E. faecium* isolates were classified as MDR. Currently, *E. faecium* and *E. faecalis* rank among the most important nosocomial pathogens worldwide and, from the epidemiological point of view, also constitute one of the most serious problems for food safety [95,96]. In accordance with the ECDC report from 2016 to 2020 on vancomycin-resistant *Enterococcus faecalis*/*E. faecium*, it was observed that the burden increased from 47,124 estimated infections, 1335 attributable deaths, and 36,542 DALYs in 2016, to 117,866 cases, 3414 attributable deaths, and 87,375 DALYs in 2020 [97]. Although virulence genes (*efaA*, *esp*, and *gelE*) and biofilm production were frequently detected (77.5% of isolates), no clear associations between virulence, biofilm capacity, and MDR were observed, consistent with previous reports [92,98,99]. Nonetheless, the high rate of biofilm formation among VRE isolates suggests enhanced environmental persistence and potential for gene transfer in slaughterhouse niches [100]. It should be noted that *Enterococcus* bacteria are less sensitive to biocidal agents than other Gram-positive bacteria, such as *Staphylococcus* spp. [101]. These observations emphasize the need for heat- or alkali-based sanitation steps and biofilm-directed detergents on high-touch equipment and containers, together with reinforced glove changing and hand hygiene protocols to limit contact-mediated transmission, and a customized measurement plan [102,103]. Vancomycin-resistant *E. faecium* was identified in one-third of *E. faecium* isolates, most frequently from carcasses, surfaces, and workers’ hands. The recovery of VRE from workers’ hands but not from nasal swabs is consistent with findings that manual contact and contaminated equipment are primary transmission routes for resistant enterococci in abattoirs [31,43]. The universal presence of the vanA gene, combined with high rates of resistance to vancomycin and teicoplanin, reflects the widespread dissemination of vanA-type VRE along the food chain [104,105,106].

Through MRSA, ESBL-*E. coli*, and VRE-*E. faecium*, the data reveal consistent patterns: the highest microbial loads occur in slaughter and evisceration zones; persistence is evident on floors, conveyors, hooks, and trays, and workers contribute measurably to transmission via their hands (and occasionally nostrils). These findings are congruent with prior occupational and environmental studies in abattoirs and argue for a multifaceted strategy: biofilm-aware sanitation using validated chemicals and mechanical action, engineering interventions to limit aerosolization and enhance hygiene zoning, and worker-focused measures including task-specific PPE, rigorous hand hygiene, and training. Collectively, such integrated approaches offer the most effective means of mitigating antimicrobial resistance risks in small-capacity slaughterhouses [10,27,102,103,107,108]. The major limitation of this study was the small number of tested low-capacity slaughterhouses. Thus, studies that examined the prevalence of MRSA, VRE-*E. faecium* and ESBL-*E. coli* in these occupational environments are sparse. The detection of the same antimicrobial-resistant pathogens, such as ESBL-producing *E. coli*, MRSA, and VRE, on carcasses, work surfaces, and among slaughterhouse workers does not in itself confirm zoonotic transmission. However, it may indicate an important epidemiological link to shared sources of exposure, including colonized animals, animal waste, and contaminated wastewater. Such findings highlight the complexity of transmission pathways in slaughterhouse environments, where direct contact with carcasses, aerosols generated during processing, and indirect exposure through contaminated equipment or waste streams may all contribute to the circulation of resistant bacteria. A critical factor amplifying these risks is the biofilm-forming capacity of antimicrobial-resistant strains. Biofilms provide a protective matrix that shields bacteria from cleaning and disinfection procedures, enables their survival on slaughterhouse surfaces and equipment, and facilitates persistence in drains, wastewater, and other niches. This not only contributes to the long-term survival of pathogens in the slaughterhouse environment but also increases the probability of co-selection between antibiotic and biocide resistance. To disentangle these relationships, more in-depth studies are required, employing high-resolution molecular techniques such as whole genome sequencing (WGS), multilocus sequence typing (MLST), or using pulsed-field gel electrophoresis (PFGE) and other source-tracking approaches [11,12,24,84,109,110]. These tools would allow for the identification of clonal relatedness between isolates from animals, environments, and humans, thereby clarifying whether observed overlaps are the result of direct zoonotic events, environmental persistence, or shared contamination reservoirs. The present study primarily aimed to demonstrate that workers in small-capacity slaughterhouses are exposed to resistant pathogens, underscoring the need to consider this risk not only within the framework of food safety monitoring but also from the perspective of occupational health and potential transmission to workers’ households. Importantly, antimicrobial-resistant bacteria and resistance genes may also originate from human-associated sources, including municipal sewage and community wastewater, which further complicates attribution and highlights the need for an integrated One Health approach [5,40]. These findings underline the urgent need for regular screening of staff, stringent enforcement of hygiene protocols, and education strategies among workers in occupational environments.

## 4. Materials and Methods

### 4.1. Study Design

The measurements were carried out in three low-capacity pork slaughterhouses having their own butcher shops, located in western and central Poland. The whole technological process was subject to strict veterinary control, and systems of good hygiene practice (GHP), good manufacturing practice (GMP), good veterinary practice (GVP), and HACCP (Hazard Analysis and Critical Control Points) were implemented. A total of 37 workers agreed to participate in the study. In each of the examined plants, bioaerosol samples, swabs from the work surfaces and meat samples were taken. Additionally, swabs from the hands and both nostrils of each participant were taken, and a questionnaire survey was conducted. The study had a positive opinion of the Bioethics Committee of the Witold Chodźko Institute of Rural Health in Lublin, Poland.

### 4.2. Collection of Biological Samples and Questionnaire Study

Prior to sampling, participants were asked to fill out questionnaires. The questionnaire provided information on gender, age, hygiene habits, job position, and occupational duties. The participants were employed at various stages of pork production, from slaughtering and cutting meat to producing ham and traditional sausages. After completing the questionnaire, a biological sample was collected from both nostrils of each participant by trained researchers wearing sterile gloves and using aseptic techniques with a flocked nylon swab dedicated to this type of research. Each swab was then placed into its plastic tube containing 1 mL of Amies medium (ESwab™ 482C, Copan, Brescia, Italy). Nasal swab collection was not conducted in people who declared injuries of the nasal mucosa, bleeding, or other medical problems that disabled sampling. Swab samples from the nostrils of workers were taken before work and after finishing the shift. The swabs from the hands of workers were taken at the end of the work shift. Swabs from workers’ hands were taken with sponges from the surfaces of both hands, from between the fingers and the outer parts of the fingers, including around the fingernails (Whirl-Pak^®^ Speci-Sponge^®^ Bags, Madison, WI, USA) in accordance with the Polish standard PN-A-82055-19:2000 [111]. After sampling, the sponges were placed in a separate Stomacher bag.

### 4.3. Bioaerosol Measurement

Air sampling was carried out stationary using the MAS impactor (Model 100 NT, Merck, Germany). The sampling time was 1 min, and the flow rate of the air was 100 L/min. After sampling at each sampling point, the device head was replaced with a new, sterilized one. The impactor was loaded with plates containing the following media (Oxoid, WB): for *Staphylococcus aureus*—agar with chromogenic substrates, Baird-Parker agar with bile and esculin (BPA); MacConkey agar (MCA)—to determine the number of *E. coli*. For enumeration of enterococci bacteria, the D-Coccosel agar (DCA, bioMérieux, Marcy-l’Étoile, France) was used.

### 4.4. Swabbing from Meat Production Line

In each tested low-capacity slaughterhouse, swabs from the production environment were taken from equipment items, machines, tools, and floors in accordance with PN-EN ISO 18593:2018-08 [112]. Swabs were collected with sponges soaked in neutralizing buffer that allows for high-efficiency recovery of microorganisms from surfaces (Whirl-Pak^®^ Hydrated Speci-Sponge^®^ Bags, Madison, WI, USA). After sampling, the sponges were placed in a separate Stomacher bag. Pork carcasses were sampled in accordance with Commission Regulation (EC) No. 1441/2007 of 5 December 2007 [113]. Swabs were taken from 4 locations of each tested carcass by a non-destructive method with an abrasive sponge (Whirl-Pak^®^ Hydrated Speci-Sponge^®^ Bags, Madison, WI, USA), and the sampling area covered 100 cm for each sampling site. The total sampling area covered 400 cm^2^. Samples taken from different carcass sites were pooled before further analysis. Samples of meat products (sausages and hams) weighing 25 g were taken using sterile instruments into a Stomacher bag for later homogenization in the laboratory in accordance with PN-ISO 6887-2 [114].

### 4.5. Microbial Analysis

In the laboratory, agar plates from the MAS impactor were incubated at 36 ± 1 °C for 24 h. After that time, the bacteria growing on the plates were enumerated to determine the number of colony-forming units (CFU) per m^3^ of sampled air. Sponges from tested surfaces and meat samples were mixed with 100 mL of peptone saline diluent (MDR, Oxoid, Basingstoke, UK, GB) and then homogenized, while swabs from the nostrils were vortexed for 30 s in transport Amies medium. After that time, ten-fold serial dilutions of each swab and sponge extract were prepared in peptone saline diluent, and then inoculated onto appropriate microbiological media for further analysis. The enumeration of *S. aureus* was carried out in accordance with PN-EN ISO 6888-1:2022-03 using Baird-Parker agar medium (Oxoid, GB) [115]. BP agar plates were incubated at 37 °C for 24 h. *E. coli* enumeration was performed on MCA and on Tryptone Bile X-glucuronide (TBX, Oxoid) according to ISO 16649-2:2001; each plate was incubated at 37 °C for 24 h aerobically [116]. In order to detect *E. faecium*, a diluted sample was spread onto DCA plates (bioMérieux, France) and incubated at 37 °C up to 48 h aerobically. Final identification of *E. coli*, *S. aureus*, and *E. faecium* isolates was performed with the commercial biochemical test: API 20E, API STAPH, and API Strep according to the producer’s instructions (bioMérieux, France).

### 4.6. Phenotypic Characterizations MRSA, ESBL-E.coli, and VRE-E. faecium

For phenotypic identification of MRSA, ESBL-*E.coli*, and VRE-*E. faecium* isolates, one loopful of each pure culture of isolated *S. aureus*, *E.coli*, and *E. faecium* was streaked onto appropriate screening media (Oxoid): Brilliance MRSA agar for detection of MRSA, Brilliance ESBL agar for ESBL-producing *E. coli*, and Brilliance VRE agar for detection of vancomycin-resistant enterococci. Detected MRSA, ESBL-*E.coli*, and VRE-*E. faecium* isolates were tested for antibiotic susceptibility using the disk diffusion method on Mueller-Hinton medium (MHA, bioMérieux SA) according to the recommendations of the EUCAST (European Committee on Antimicrobial Susceptibility Testing) (2025) [117]. For MRSA isolates, the following antibiotic disks were used (Oxoid, GB): cefoxitin (FOX, 30 μg), clindamycin (DA, 2 μg), ciprofloxacin (CIP, 5 μg), erythromycin (E,15 μg), fusidic acid (FD,10 μg), gentamicin (CN, 10 μg), kanamycin (K 30 μg), linezolid (LNZ,10 μg), penicillin (P, 10U), quinupristin–dalfopristin (QD, 15 μg), tetracycline (TE, 30 μg), and rifampicin (RD, 30 μg). The vancomycin-resistant *E. faecium* was tested for susceptibility to ampicillin (AMP, 10 µg), imipenem (IPM, 10 µg), gentamicin (CN, 30 µg), ciprofloxacin (CIP, 5 µg), levofloxacin (LVX, 5 µg), erythromycin (E, 15 µg), quinupristin–dalfopristin (QDA, 15 µg), sulfamethoxazole–trimethoprim (SXT, 1.25–23.75 µg), linezolid (LNZ, 10 µg), nitrofurantoin (F, 100 µg), vancomycin (VAN, 5 µg), and teicoplanin (TEC, 30 µg). ESBL production in *E. coli* isolates was confirmed phenotypically by the double disk synergy test (DDST) and the combined disk diffusion test, according to the EUCAST [117]. The isolates were tested to evaluate the pattern of antimicrobial susceptibilities to ampicillin (AMP, 10 ug), piperacillin (PRL, 30 ug), ceftazidime (CAZ, 30 μg), cefotaxime (CTX, 30 μg), nalidixic acid (NA, 30 μg), cefuroxime (CXM, 30 μg) cefepime (FEP, 30 μg), imipenem (IMP, 10 μg), meropenem (MEM, 10 μg), amikacin (AK, 30 μg), gentamicin (CN, 10 μg) tobramycin (TOB, 10 μg), ofloxacin (OFX, 5 μg), fosfomycin (FF, 200 μg), and tetracycline (TET, 30 µg). Multidrug resistance (MDR) in isolates was identified when they exhibited resistance to at least one agent in three or more tested antimicrobial categories [118]. The multiple antibiotic resistance index (MARI) was calculated by dividing the total number of antimicrobials to which the isolate was resistant by the total number of antimicrobials tested against the isolate [119]. For quality control, the control strains were used: *S. aureus* ATCC 25923—methicillin-sensitive, *S. aureus* ATCC 43300—methicillin-resistant, *E. faecalis* ATCC 29212—glycopeptide-sensitive, *E. faecalis* ATCC 51299—glycopeptide-resistant, *E. coli* ATCC 25922—negative control for ESBL, and *K. pneumoniae* ATCC 700603—positive control for ESBL. The confirmed samples of MRSA, ESBL, and VRE were stored at −80 °C and were subjected to further genetic testing.

### 4.7. Detection of Biofilm-Forming Abilities of MRSA, ESBL-E.coli, and VRE-E. faecium

Biofilm formation of isolated MRSA, ESBL-*E.coli*, and VRE-*E. faecium* strains was examined using a microplate method [31]. Briefly, 20 µL of bacterial suspension in sterile saline (10^8^ cfu/mL) was added to 180 µL of TSB medium with 1% glucose (Oxoid) in a 96-well microplate in 8 replicates, and incubated 24 h at 36 °C. The positive control was a suspension with the biofilm-forming strain *S. epidermidis* ATCC 35984, while the negative control wells contained broth only—200 µL of TSB supplemented with 1% glucose per well. After incubation, microplate wells were rinsed 3 times with sterile PBS buffer and then placed in an incubator at 60 °C for 60 min to fix the adhered cells. Next, 150 µL of crystal violet was applied to each well, and the plate was incubated for 15 min at room temperature. After another washing, 150 µL of ethanol was applied to each well, and the plate was again incubated for 30 min. After this time, the optical density (OD) was measured at 570 nm using the iMark microplate reader (BioRad, Hercules, CA, USA). The cut-off value (ODc) was derived from 3 standard deviations above the mean of the negative control. Strains were classified as follows: OD < ODc—non-biofilm formers; ODc < OD < 2× ODc—weak biofilm formers; 2× ODc < OD—moderate biofilm formers; 4× ODc < OD—strong biofilm formers.

### 4.8. Genotypic Characterizations of MRSA, ESBL-E.coli, and VRE-E. faecium

After phenotypic identification, the collected MRSA, VRE-*E. faecium*, and ESBL-*E.coli* isolates were investigated by polymerase chain reaction (PCR) based on amplification of selected genes targeting genes determining antibiotic resistance, biofilm-forming abilities, and antiseptic resistance. For this purpose, total genomic DNA of the bacterial isolates was extracted using the Genomic Mini DNA kit (A&A Biotechnology, Gdynia, Poland) according to the manufacturer’s instructions. All DNA extracts were stored at −20 °C before PCR. The sequences of the primers, the sizes of the PCR products, and the corresponding references are summarized in Table 4.

In brief, amplification reactions were set in a 25 µL volume containing 12.5 µL of PCR master mix (contains Mg++, dNTPs, and recombinant Taq DNA polymerase 0.1 U/µL), 0.5 µL (10 pmol) of each of the forward and reverse primers, 2 µL of DNA, and nuclease-free water (A&A Biotechnology, Gdansk, Poland). All PCR amplifications were performed using optimized conditions described in the corresponding published studies (Table 4), employing a C1000 Thermal Cycler (Bio-Rad, Hercules, CA, USA). The reactions followed the same thermal profile: an initial denaturation at 95 °C for 5 min; 35 amplification cycles consisting of 40 s at 95 °C, 50 s at gene-specific annealing temperatures, and 50 s at 72 °C; followed by a final extension at 72 °C for 10 min. The PCR amplicons were confirmed by gel electrophoresis on a 1.5% agarose gel and visualized under UV light.

MRSA isolates were tested by duplex PCR targeting genes determining methicillin resistance (*mecA* and *mecALGA251/mecC*) [131]. All MRSA isolates were screened for the detection of biofilm genetic determinants such as *icaA*, *icaBC*, *fnbA*, *fnbB*, and *cna* genes [123,124], and antiseptic resistance genes encoding multidrug efflux pumps such as *qacAB*, *smr*, and *qacG* [120,121]. Detection of ESBL genes was confirmed by multiplexing the three ESBL genes *blaCTX-M*, *blaTEM*, and *blaSHV* [134]. The ESLB isolates were tested for carriage of the genetic biofilm determinants, including those coding for type 1 fimbriae (*fimH*) [128], adhesin type 3 pili (*mrkD*) [127], and S and F1C fimbriae (*sfa* and *foc*) [129], as well as quaternary ammonium compounds (QACs) resistance genes such as *ydgF*, *qacEΔ1*, *emrE*, and *mdfA* [130]. VRE isolates of *E. faecium* were tested for the presence of *vanA* and *vanB* genes [132,133]. For each VRE-*E. faecium* isolate, the biofilm-forming genes such as enterococcal surface protein (*esp*), gelatinase (*gelE*), cell wall adhesins (*efaA*), and antiseptic resistance genes such as *qacAB*, *smr*, and *qacG* were amplified using specific primers previously described [121,125].

### 4.9. Data Analyses

The relationship between the occurrence of antibiotic resistance, biofilm-forming abilities, and gene carriers was assessed using the chi-square test. All statistical analyses were performed using Statistica software (IBM Corp., Armonk, New York, NY, USA), and a *p*-value < 0.05 was considered statistically significant.

## 5. Conclusions

The occurrence of MRSA, ESBL-*E. coli*, and VRE-*E. faecium* in low-capacity slaughterhouses highlights the role of these facilities as reservoirs and transmission points for multidrug-resistant bacteria. The detection of resistance and biofilm-associated genes across all three taxa underscores the urgent need for targeted interventions, including validated sanitation programs that focus on biofilm reservoirs, improved air quality control, and enhanced enforcement of occupational hygiene. The integration of molecular surveillance for *mecA*, *blaCTX-M*, and *vanA* genes, along with antimicrobial resistance markers such as *qacA/B* and *qacEΔ1*, will be crucial for the early detection and control of resistant pathogens in the meat-processing sector. Our findings indicate that slaughterhouse workers are frequently exposed to antibiotic-resistant bacteria, including MRSA, ESBL-*E. coli*, and VRE-*E. faecium*. The highest risks occur in slaughter and evisceration areas, where contaminated air, carcasses, and surfaces create reservoirs for these pathogens. Workers in these zones are not only more likely to carry resistant bacteria themselves but can also spread them to meat products and, eventually, to consumers. This issue is significant for two reasons: it poses a risk to workers of contracting difficult-to-treat infections, and it contributes to the broader public health issue of antimicrobial resistance. To mitigate these risks, small slaughterhouses should implement stronger hygiene measures that focus on biofilms, maintain better separation between clean and dirty areas, enhance ventilation, and strictly enforce the use of protective equipment. Regular training and monitoring of workers are also essential. Protecting employees in this way will help protect consumers and limit the spread of resistance.

## Figures and Tables

**Table 1:** Characteristics of the studied population of workers.

Demographics	Butchers, *n* = 30	Non-Production Workers, *n* = 7
Age: mean (SD)	42.4 (12.2)	37.1 (7.8)
Female (%)	3 (9.7)	5 (71.4)
Use of antibiotics (within 6 months)	2 (6.7%)	1 (14.3%)
Living on an agricultural or livestock farm	4 (13.3%)	1 (14.3%)
Length of employment in current position (years)	8.3 (7.1)	7.7 (9.4)

**Table 2:** Proportion of positive samples for ESBL-*E. coli*, MRSA, and VRE-*E. faecium* bacteria.

Sampling Sources (No. of Samples)	ESBL-*E. coli*		MRSA		VRE-*E. faecium*	
	*n*	%	*n*	%	*n*	%
**Slaughter and evisceration sections**						
Bioaerosol (*n* = 4)	3	75.0%	1	25.0%	1	25.0%
Conveyors (*n* = 8)	2	25.0%	2	25.0%	3	37.5%
Hooks and trays (*n* = 9)	6	66.7%	3	33.3%	2	22.2%
Saws (*n* = 8)	1	12.5%	1	12.5%	2	25.0%
Floors (*n* = 8)	3	37.5%	3	37.5%	4	50.0%
**Meat cutting sections**						
Bioaerosol (*n* = 6)	3	50.0%	0	0.0%	0	0.0%
Knives and cleavers (*n* = 10)	1	10.0%	0	0.0%	1	10.0%
Work tops (*n* = 10)	2	20.0%	1	10.0%	2	20.0%
Containers	3	37.5%	3	37.5%	2	25.0%
Floors	1	12.5%	2	25.0%	2	25.0%
**Meat processing sections**						
Bioaerosol (*n* = 10)	3	30.0%	1	10.0%	0	0.0%
Grinders (*n* = 9)	3	33.3%	1	11.1%	2	22.2%
Stuffer machines (*n* = 8)	1	12.5%	3	37.5%	2	25.0%
Containers (*n* = 9)	2	22.2%	2	22.2%	0	0.0%
Work tops (*n* = 9)	1	11.1%	1	11.1%	0	0.0%
Floors (*n* = 9)	0	0.0%	2	22.2%	4	44.4%
**Warehouse/retail space**						
Bioaerosol (*n* = 6)	0	0.0%	0	0.0%	0	0.0%
Work tops (*n* = 8)	0	0.0%	0	0.0%	1	12.5%
Butcher scales (*n* = 7)	1	14.3%	0	0.0%	0	0.0%
Floors (*n* = 7)	1	14.3%	1	14.3%	1	14.3%
**Meat**						
Carcasses (*n* = 15)	8	53.3%	7	46.7%	5	33.3%
Ready-to-eat product (*n* = 18)	0	0.0%	0	0.0%	0	0.0%
**Hands swabs**						
Butchers (*n* = 30)	6	20.0%	1	3.3%	5	16.7%
Non-production workers (*n* = 7)	0	0.0%	1	14.3%	1	14.3%
**Nasal swabs**						
Butchers	3	10.0%	1	3.3%	0	0.0%
Non-production workers	1	14.3%	0	0.0%	0	0.0%

**Table 3:** Prevalence of ESBL-*E. coli*, MRSA, VRE-*E. faecium*, and antibiotic-sensitive isolates (non-ESBL-*E. coli*, MSSA, and VSE-*E. faecium*) collected in the studied low-capacity slaughterhouse.

Sampling Sources (No. of Samples)	Non- ESBL-*E. coli* *n* (%)	ESBL-*E. coli* *n* (%)	MSSA *n* (%)	MRSA *n* (%)	VSE- *E. faecium* *n* (%)	VRE- *E. faecium* *n* (%)
Bioaerosols (*n* = 26)	12 (57%)	9 (43%)	13 (87%)	2 (13%)	13 (93%)	1 (7%)
Equipment and surfaces (*n* = 135)	60 (68%)	28 (32%)	73 (74%)	25 (26%)	43 (61%)	28 (39%)
Pork carcasses (*n* = 15)	4 (33%)	8 (67%)	3 (30%)	7 (70%)	7 (58%)	5 (42%)
Ready-to-eat meat products (*n* = 18)	6 (100%)	0 (0%)	5 (100%)	0 (0%)	5 (100%)	0 (0%)
Hand swabs (*n* = 37)	28 (82%)	6 (18%)	18 (90%)	2 (10%)	8 (57%)	6 (43%)
Nasal swabs (*n* = 37)	15 (79%)	4 (21%)	5 (83%)	1 (17%)	5 (100%)	0 (0%)

**Table 4:** The primers used for the detection of genes involved in antibiotic resistance, biofilm formation, and resistance to quaternary ammonium compounds.

Gene	Primer Sequences (5′-3′)	Product Size (bp)	References
*qacA/B*	GCAGAAAGTGCAGAGTTCG CCAGTCCAATCATGCCTG	361	[120,121]
*smr*	GCCATAAGTACTGAAGTTATTGGA GACTACGGTTGTTAAGACTAAACCT	195	[120,121]
*qacG*	CAA CAG AAA TAA TCG GAA CT TAC ATT TAA GAG CAC TAC A	275	[121,122]
*fnbpB*	TCTGCGTTATGAGGATTT ACAGTAGAGGAAAGTGGG	452	[123]
*fnbpA*	TCCGCCGAACAACATACC TCAAGCACAAGGACCAAT	952	[123]
*cna*	CGATAACATCTGGGAATAAA ATAGTCTCCACTAGGCAACG	716	[123]
*icaBC*	GCCTATCCTTATGGCTTGA TGGAATCCGTCCCATCTC	182	[123]
*icaA*	ACACTTGCTGGCGCAGTCAA TCTGGAACCAACATCCAACA	188	[124]
*gelE*	AGTTCATGTCTATTTTCTTCAC CTTCATTATTTACACGTTTG	402	[125]
*efaA*	CGTGAGAAAGAAATGGAGGA CTACTAACACGTCCACGAATG	499	[125]
*esp*	TTACCAAGATGGTTCTGTAGGCAC CCAAGTATACTTAGCATCTTTTGG	913	[126]
*mrkD*	CCACCAACTATTCCCTCGAA ATGGAACCCACATCGACATT	228	[127]
*fimH*	GAGAAGAGGTTTGATTTAACTTATTG AGAGCCGCTGTAGAACTGAGG	559	[128]
*sfa/focDE*	CTCCGGAGAACTGGGTGCATCTTAC CGGAGGAGTAATTACAAACCTGGCA	410	[129]
*ydgF*	TAGGTCTGGCTATTGCTACGG GGTTCACCTCCAGTTCAGGT	330	[130]
*qacEΔ1*	AATCCATCCCTGTCGGTGTT CGCAGCGACTTCCACGATGGGGAT	175	[130]
*emrE*	TATTTATCTTGGTGGTGCAATAC ACAATACCGACTCCTGACCAG	195	[130]
*mdfA*	GCATTGATTGGGTTCCTAC CGCGGTGATCTTGATACA	284	[130]
*mecA*	TCCAGATTACAACTTCACCAGG CCACTTCATATCTTGTAACG	162	[131]
*mecC* (*mecALGA251*)	GAAAAAAAGGCTTAGAACGCCTC GAAGATCTTTTCCGTTTTCAGC	138	[131]
*vanA*	GGGAAAACGACAATTGC GTACAATGCGGCCGTTA	732	[132]
*vanB*	ACCTACCCTGTCTTTGTGAA AATGTCTGCTGGAACGATA	300	[133]
*blaTEM*	TTGGGTGCACGAGTGGGTTA TAATTGTTGCCGGGAAGCTA	506	[134]
*blaSHV*	TCGGGCCGCGTAGGCATGAT AGCAGGGCGACAATCCCGCG	628	[134]
*blaCTX-M*	ATGTGCAGYACCAGTAARGTKATGGC TGGGTRAARTARGTSACCAGAAYSAGCGG	592	[134]

## Data Availability

The data that support the findings of this study are available from the corresponding author upon reasonable request.

## Supplementary Materials

The following supporting information can be downloaded at: https://www.mdpi.com/article/10.3390/antibiotics14121200/s1. Table S1: Frequency of antimicrobial resistance profiles of ESBL-*E. coli* isolates from tested low-capacity slaughterhouses; Table S2: Frequency of antimicrobial resistance profiles of MRSA isolates from tested low-capacity slaughterhouses; Table S3: Frequency of antimicrobial resistance profiles of VRE-*E. faecium* isolates from tested low-capacity slaughterhouses.

## Abbreviations

The following abbreviations are used in this manuscript:

ESBL	Extended-Spectrum Beta-Lactamases
MARI	Multiple Antibiotic Resistance Index
MDR	Multidrug Resistance
MRSA	Methicillin-Resistant *Staphylococcus aureus*
MSSA	Methicillin-Sensitive *Staphylococcus aureus*
non-ESBLs	Isolates that do not produce ESBL enzyme
VRE	Vancomycin-Resistant *Enterococcus*
VSE	Vancomycin-Sensitive *Enterococcus*
