# Supplementary material for: Prevalence of Methicillin-Resistant S. aureus, Extended-Spectrum β-Lactamase-Producing E. coli, and Vancomycin-Resistant E. faecium in the Production Environment and Among Workers in Low-Capacity Slaughterhouses in Poland
Source: Antibiotics (Basel). 2025 Nov 28;14(12):1200. doi: 10.3390/antibiotics14121200 (PMC12729451; doi:10.3390/antibiotics14121200)
Supplement: Supplementary file 1 [file antibiotics-14-01200-s001.zip › antibiotics-3972516-supplementary.pdf]

Table S1. Frequency of antimicrobial resistance profiles of ESBL-*E. coli* isolates from tested low-capacity slaughterhouses.

Antimicrobial resistance profiles*	Workers	Bioaerosol	Surfaces and equipment	Carcasses	Total	MARI
AM-AMC-PRL-CTX-CAZ-CXM-FEP	2	1	7		**10**	0.44
AM-AMC-PRL-CTX-CAZ-CXM-FEP-OFX-NA-FF-TE		1	2	1	**4**	0.69
AM-AMC-PRL-CTX-CAZ-CXM-FEP-OFX-FF		1	1	1	**3**	0.56
AM-AMC-CTX-CAZ-CXM		1	1		**2**	0.31
AM-AMC-PRL-CTX-CAZ-CXM-FEP-AK-TE	2				**2**	0.56
AM-AMC-PRL-CTX-CAZ-CXM-FEP-FF	1	1			**2**	0.50
AM-AMC-PRL-CTX-CAZ-CXM-FEP-OFX-NA			2		**2**	0.56
AM-AMC-PRL-CTX-CAZ-CXM-FEP-OFX-NA-CN-TOB			2		**2**	0.69
AM-AMC-PRL-CTX-CAZ-CXM-FEP-OFX-NA-FF			2		**2**	0.63
AM-AMC-PRL-CTX-CAZ-CXM-FEP-TE				2	**2**	0.50
AM-AMC-PRL-CTX-CAZ-CXM-OFX				2	**2**	0.44
AM-AMC-CTX-CAZ-CXM-FEP-OFX-NA-AK-TOB			1		**1**	0.63
AM-AMC-CTX-CAZ-CXM-FEP-OFX-NA-FF			1		**1**	0.56
AM-AMC-PRL-CTX-CAZ-CXM			1		**1**	0.38
AM-AMC-PRL-CTX-CAZ-CXM-AK-TE			1		**1**	0.50
AM-AMC-PRL-CTX-CAZ-CXM-FEP-AK	1				**1**	0.50
AM-AMC-PRL-CTX-CAZ-CXM-FEP-AK-FF	1				**1**	0.56
AM-AMC-PRL-CTX-CAZ-CXM-FEP-CN-TOB				1	**1**	0.56
AM-AMC-PRL-CTX-CAZ-CXM-FEP-CN-TOB-FF-TE	1				**1**	0.69
AM-AMC-PRL-CTX-CAZ-CXM-FEP-IM-MEM			1		**1**	0.56
AM-AMC-PRL-CTX-CAZ-CXM-FEP-IM-OFX-FF				1	**1**	0.63
AM-AMC-PRL-CTX-CAZ-CXM-FEP-NA-TOB		1			**1**	0.56
AM-AMC-PRL-CTX-CAZ-CXM-FEP-NA-TOB-FF		1			**1**	0.63
AM-AMC-PRL-CTX-CAZ-CXM-FEP-OFX-AK-FF-TE				1	**1**	0.69
AM-AMC-PRL-CTX-CAZ-CXM-FEP-OFX-FF-TE		1			**1**	0.63
AM-AMC-PRL-CTX-CAZ-CXM-FEP-OFX-NA-AK-TOB-TE	1				**1**	0.75
AM-AMC-PRL-CTX-CAZ-CXM-FEP-OFX-NA-CN				1	**1**	0.63
AM-AMC-PRL-CTX-CAZ-CXM-FEP-OFX-NA-CN-TE		1			**1**	0.69
AM-AMC-PRL-CTX-CAZ-CXM-FEP-OFX-NA-CN-TOB-FF-TE			1		**1**	0.81
AM-AMC-PRL-CTX-CAZ-CXM-FEP-OFX-NA-TOB-FF-TE	1				**1**	0.75
AM-AMC-PRL-CTX-CAZ-CXM-OFX-NA			1		**1**	0.50
AM-AMC-PRL-CTX-CAZ-CXM-OFX-NA-AK-TE			1		**1**	0.63
AM-AMC-PRL-CTX-CAZ-CXM-TE			1		**1**	0.44

AM (ampicillin), AMC (amoxicillin–clavulanic acid), PRL (piperacillin), CTX (cefotaxime), CAZ (ceftazidime), CXM (cefuroxime), FEP (cefepime), IMP (imipenem), MEM (meropenem), OFX (ofloxacin), NA (nalidixic Acid), AK (amikacin), CN (gentamicin), TOB (tobramycin), FF (nitrofurantoin), TE (tetracycline)

Table S2. Frequency of antimicrobial resistance profiles of MRSA isolates from tested low-capacity slaughterhouses.

Antimicrobial resistance profiles*	Workers	Bioaerosol	Surfaces and equipment	Carcasses	Total	MARI
FOX-CIP			4		**4**	0.17
FOX-ERY-DA-CIP	1		2		**3**	0.33
FOX-ERY-DA-TE-CIP-RD			2	1	**3**	0.50
FOX-ERY-FD			2	1	**3**	0.25
FOX-K-TOB-ERY-DA-CIP		1	1		**2**	0.50
FOX-K-TOB-GEN-ERY-DA		1	1		**2**	0.50
FOX-K-TOB-GEN-ERY-DA-CIP			2		**2**	0.58
FOX-QD-CIP			1	1	**2**	0.25
FOX-GEN-DA-CIP				1	**1**	0.33
FOX-GEN-ERY-DA	1				**1**	0.33
FOX-GEN-ERY-DA-TE-CIP-RD				1	**1**	0.58
FOX-GEN-ERY-TE			1		**1**	0.33
FOX-GEN-ERY-TE-FD			1		**1**	0.42
FOX-GEN-RD			1		**1**	0.25
FOX-K-ERY			1		**1**	0.25
FOX-K-ERY-DA			1		**1**	0.33
FOX-K-GEN-ERY			1		**1**	0.33
FOX-K-GEN-ERY-LNZ			1		**1**	0.42
FOX-K-TOB-GEN-CIP				1	**1**	0.42
FOX-K-TOB-GEN-CIP-RD				1	**1**	0.50
FOX-K-TOB-GEN-ERY-DA-RD			1		**1**	0.58
FOX-TE-CIP	1				**1**	0.25
FOX-TOB-ERY-DA-TE-CIP			1		**1**	0.50
FOX-TOB-GEN-ERY-QD-TE			1		**1**	0.50

FOX (cefoxitin), K (kanamycin), TOB (tobramycin), GEN (gentamicin), ERY (erythromycin), DA (clindamycin), QD (quinupristin–dalfopristin), TE (tetracycline), CIP (ciprofloxacin), LNZ (line-zolid), FD (fosfomycin), RD (rifampicin)

Table S3. Frequency of antimicrobial resistance profiles of VRE-*E. faecium* isolates from tested low-capacity slaughterhouses.

Antimicrobial resistance profiles*	Workers	Bioaerosol	Surfaces and equipment	Carcasses	Total	MARI
AP-CIP-LEV-QD-VA-TEC-			2	1	3	0.50
AP-CIP-LEV-VA-TEC-	1		2		3	0.42
AP-IPM-VA-TEC-			3		3	0.33
GEN-VA-TEC-			3		3	0.25
AP-CIP-QD-VA-TEC-	1		1		2	0.42
CIP-LEV-VA-TEC	1		1		2	0.33
GEN-ERY-VA-TEC-			2		2	0.33
AP-CIP-LEV-QD-VA-TEC-			1		1	0.50
AP-CIP-LEV-ERY-VA-TEC-			1		1	0.50
AP-CIP-QD-F-VA-TEC-			1		1	0.50
AP-GEN-CIP-LEV-ERY-VA-TEC-				1	1	0.58
AP-GEN-CIP-VA-TEC				1	1	0.42
AP-GEN-VA-TEC-				1	1	0.33
AP-IPM-CIP-LEV-QD-VA-TEC-			1		1	0.58
AP-IPM-CIP-LNZ-VA-TEC-			1		1	0.50
AP-IPM-ERY-F-VA-TEC-		1			1	0.50
AP-IPM-GEN-ERY-VA-TEC-				1	1	0.50
AP-IPM-GEN-LEV-VA-TEC-			1		1	0.50
AP-LNZ-VA-TEC-	1				1	0.33
CIP-ERY-VA-TEC-	1				1	0.33
CIP-LEV-ERY-QD-VA-TEC-			1		1	0.50
CIP-LEV-ERY-VA-TEC-	1				1	0.42
CIP-QD-LNZ-VA-TEC-		1			1	0.42
CIP-VA-TEC-			1		1	0.25
ERY-VA-TEC-			1		1	0.25
GEN-CIP-VA-TEC-			1		1	0.33
IPM-CIP-LEV-VA-TEC-			1		1	0.42
IPM-GEN-ERY-VA-TEC-			1		1	0.42
LEV-SXT-VA-TEC-			1		1	0.33

*AP (ampicillin), IPM (imipenem), GEN (gentamicin), CIP (ciprofloxacin), LEV (levofloxacin), ERY (erythromycin), QD (quinupristin–dalfopristin), SXT (trimethoprim–sulfamethoxazole), LNZ (linezolid), F (nitrofurantoin), VA (vancomycin), TEC (teicoplanin).
